# Sequential Bayesian-optimized graphene synthesis by direct solar-thermal chemical vapor deposition

**DOI:** 10.1038/s41598-024-54005-z

**Published:** 2024-02-13

**Authors:** Abdalla Alghfeli, Timothy S. Fisher

**Affiliations:** Mechanical and Aerospace Engineering Department, University of California Los Angeles, 420 Westwood Plaza, Los Angeles, CA 90095 USA

**Keywords:** Synthesis of graphene, Devices for energy harvesting

## Abstract

This work reports the use of a high-flux solar simulator that mimics the solar spectrum and a cold-wall CVD reactor to demonstrate the feasibility of utilizing a renewable energy resource in synthesizing graphene under various conditions. A parametric study of process parameters was conducted using a probabilistic approach. Gaussian process regression serves as a surrogate to establish a prior for Bayesian optimization, and an information acquisition function is employed to identify conditions that yield high-quality products. Backscattered electron images and Raman mapping were used to assess the effects of growth conditions on graphene characteristic sizes, film quality, and uniformity. We report the synthesis of high-quality single-layer graphene (SLG) and AB-stacked bilayer graphene films in a one-step, short-time process with $I_{D}/I_{G}$ ratios of 0.21 and 0.14, respectively. Electron diffraction analysis shows peak intensities that resemble SLG and AB-bilayer graphene with up to 5 and 20 ${\upmu }$m grain sizes, respectively. The optical transmissivities of SLG and AB-bilayer graphene fall between 0.959–0.977 and 0.929–0.953, whereas the sheet resistances measured by a 4-point probe with 1 mm spacing are 15.5 ± 4.6 and 3.4 ± 1.5 k$\Omega$/sq, respectively. Further scale-up of the optimized graphene growth area was achieved by flattening the insolation profile, leading to spatial uniformity up to 13 mm in radius. Direct solar capture for CVD synthesis enable a practical and sustainable option for synthesizing graphene films applicable for photonic and electronic applications.

## Introduction

Graphene is a two-dimensional material that has attracted much attention due to its extraordinary properties such as high electrical and thermal conductivities^1–3^, excellent transparency^4^, and chemical stability^5^. Such properties make graphene a compelling material candidate in photonic and electronic applications such as transparent conductive electrodes with high mechanical flexibility^6,7^ and strength^8^ for solar cells^5,6^, supercapacitors^9–11^, and lithium-ion batteries^11^. In addition to the superior properties of a single graphene layer (SLG), Bernal-stacked (AB) bilayer graphene provides a tunable bandgap by an external electric field suitable for field-effect transistors^12,13^. Many techniques have been developed to scale-up graphene production by liquid phase^14^ and shear mixing^15^ graphite exfoliation to accommodate industrial needs.

Graphene synthesis by chemical vapor deposition (CVD)^16^, another synthesis approach, utilizes heating sources from either plasma or electric heaters through a hot-wall furnace or cold-wall reactor. However, these approaches consume much energy and can harm the environment. More broadly, studies indicate that semiconductor fabrication consumes up to 100 MWh^17^ of electricity hourly, where 0.386 kg of CO$$_{2}$$ is emitted per kWh^18^. Related energy expenses have been reported to account for up to 30% of the operating costs^17,19^. Additionally, as detailed in Pedersen’s 2021 study^20^, the estimated electrical energy consumption and CO$$_{2}$$ emissions for the production of 300 mm wafers were reported as 1400 kWh/wafer and 300 kg/wafer, respectively. Assuming the cost of electricity at 22.33 cents/kWh^21^, the calculated cost per wafer would be approximately $300. Moreover, factoring in the utilization of solar energy to ensure a process free of CO$$_{2}$$ emissions to the environment, integrating heat generation, which would consume the majority of the required energy, from renewable resources could significantly diminish overall costs. Here, we report the use of a high flux solar simulator (HFSS) that mimics the solar spectrum and a cold-wall CVD reactor to achieve graphene synthesis under variable conditions. Our process optimization utilizes a Bayesian–Gaussian surrogate model to navigate through various conditions and to optimize graphene quality.

In typical CVD reactors, the heater and electronic instruments typically consume around 3–3.5 kW of power, although plasma technology can decrease the required power as noted in a prior study^20^. However, additional energy is necessary for supplementary heating and plasma processes. When considering the power ratings of various solar-thermal CVD equipment, the entire process, including substrate annealing, takes 20 min. By utilizing concentrating mirrors to harness solar energy in a potential field test, a heating power of 2.5 kW would facilitate graphene synthesis, consuming less than 1 kWh of solar energy. Previous research efforts have predominantly relied on hot furnaces and substrate heaters for AB bilayer graphene synthesis, involving lengthy processing times of up to 7 h. The total energy consumption per unit area in these studies was approximately $10^{4}$ kW h m$^{-2}$, significantly higher than the 700 kW h m$^{-2}$ achieved through solar-thermal CVD in the current study.

Many prior studies have used plasma CVD to grow uniform SLG films^22,23^, free-standing graphene layers (FGL)^24^, and carbon nanostructures for supercapacitors such as polygonal carbon nanofibers^10^ and bioinspired micro-conduits^9^. The advantage of plasma CVD is that it enables synthesis at low temperatures of 300–700 $^{\circ }$C on non-catalytic materials^23,24^. Hot-wall^13,25–29^ and cold-wall^30–35^ reactor CVD using electrical sources to heat either the entire flow or the substrate locally have also demonstrated the ability to synthesize high quality SLG and AB-stacked bilayer graphene films. The advantages of CVD synthesis include control of graphene quality, number of layers, and stacking orientation through growth kinetics and thermodynamics^25–28,36,37^, as well as scaling up the production by roll-to-roll processing^23,38^.

Some have utilized CVD mechanisms to exploit the effect of temperature on the kinetics that govern nucleation, grain growth, and film thickness^25,29^. Others have modulated vacuum pressure and catalyst substrate solubility to improve thickness uniformity and defect density^26^. Hydrogen concentration has been found to influence grain sizes and number of layers because it serves as an etchant and activator for carbon bounds^27^. Such studies^13,25–28^ have demonstrated that graphene synthesis at high temperatures, low pressures, and high H$_{2}$ concentrations (low CH$_{4}$:H$_{2}$ ratio) generally lead to higher graphene quality, larger grain sizes, reduced nucleation density, and uniform (single or bilayer) films. Further, simulations of graphene growth have been conducted through a validated COMSOL model to study growth thermodynamics, kinetics in the gas phase, and surface reactions^36^. Additionally, kinetic models have been derived to assess graphene growth on different transition metal catalysts such as Co, Ni, and Cu while taking into account carbon permeation effects, process thermodynamics, and carbon solubility^33^.

Statistical techniques such as factorial designs^39^ are less efficient in finding the optimal conditions for graphene synthesis due to their complexity, especially while studying a large number of parameters. To achieve stochastic optimization of the $I_{D}/I_{G}$ Raman peak ratio, a Gaussian process regression (GPR) is employed here as a surrogate model to determine a prior for Bayesian optimization (BO) and an acquisition function that quantifies the model information^38,40–43^. We demonstrate the use of a HFSS and a cold-wall CVD reactor to execute a parametric study on graphene growth parameters. Such a heating source offers the ability to harvest the sun’s renewable energy to heat the sample locally in a short time and without substrate contact to synthesize high-quality SLG and AB-stacked bilayer graphene films.

## Experimental setup and methods

### Solar-thermal CVD system

A custom-built high flux solar simulator (HFSS) characterized in previous work^44^ is used here, and initial observations of predominant AB-stacking have been reported^45^. The HFSS is equipped with a 10 kW$_{e}$ short xenon arc lamp (Superior Quartz, SQP-SX100003) that approximates the solar spectrum and a truncated ellipsoidal reflector (Optiforms, E1023F). The heat flux irradiation on the target has been characterized with a Gaussian–Lorentzian-like profile as:

1 $$\begin{aligned} q^{\prime \prime }(r, I)=A(I)\left[ \frac{1-\alpha }{\sigma _{g} \sqrt{2 \pi }} e^{-r^{2} / 2 \sigma _{g}^{2}}+\frac{\alpha }{\pi }\left( \frac{\sigma _{l}}{r^{2}+\sigma _{l}^{2}}\right) \right] \end{aligned}$$

where *A*(*I*) = (0.740 $\times I$ - 20.5) kW/m, $\sigma _{l}$ = 0.0492 m, $\sigma _{g}$ = 0.00829 m, and $\alpha$ = 0.519^44^. By varying the current (*I*) of a DC power supply in the (100–200 A) range, the output heat flux can be controlled precisely.

A custom-built CVD system equipped with a metered gas supply, vacuum pump, and water-cooled stainless steel reactor with a 25.4 cm quartz viewport has been integrated with the HFSS in previous work^46^. The system includes mass flow controllers (MKS, GM50A) calibrated for CH$_{4}$ and H$_{2}$ with flow rates up to 100 and 1000 sccm, respectively, a capacitance manometer (MKS, 624F) in the range of 1000 torr, a rotary vane vacuum pump (Oerlikon-Leybold, D65BCS), and a control throttle valve (MKS, T3BI). Additionally, a single wavelength (5 ${\upmu }$m) pyrometer (Williamson, SP-GL-20C) is used to acquire temperature measurements from the copper substrate remotely.

**Figure 1:**
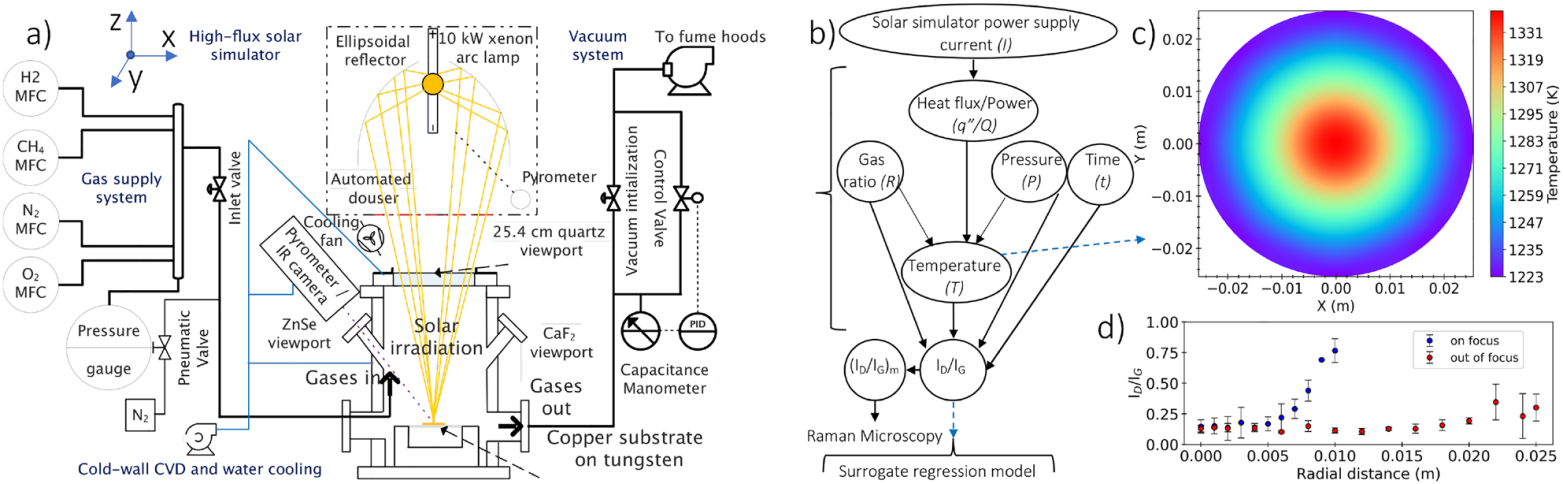
(**a**) Overview of solar-thermal CVD setup for graphene synthesis. (**b**) Flow chart for graphene synthesis parameters. (**c**) Copper substrate temperature profile prior to graphene growth computed by a validated numerical thermal model at HFSS current of 107 A and vacuum pressure of 10 torr (under hydrogen atmosphere) with the lamp in the focal plane. (**d**) Comparison between spatial uniformity ($I_{D}/I_{G}$) of graphene on Cu when the lamp is in and out of the focal plane, where the heat flux profile is flattened when the lamp is out of its focal plane to achieve temperature uniformity on a larger area.

A related numerical heat transfer model has been developed and validated in previous work^46^ for a stationary circular copper substrate (50.8 mm in diameter and 76 ${\upmu }$m thick). To facilitate the synthesis process, the model predicts the substrate temperature profile at various HFSS current and vacuum pressure conditions prior to graphene growth. Further scale-up of synthesized graphene at optimized conditions can be achieved by displacing the lamp 4.8 mm towards the target, out of its focal plane, to flatten the heat flux, achieving a more uniform profile. Therefore, a larger area on the copper substrate reaches a uniform temperature of 1060 $\pm \,10\,^{\circ }$C but at the expense of more power consumed by the HFSS (current at 155 A). Based on Raman measurements ($I_{D}/I_{G}$ ratio), spatial uniformity up to 13 mm in radius is achieved, representing an order of magnitude increase in synthesized area. Figure 1 illustrates an overview of solar-thermal CVD setup, a flow chart for synthesis parameters, a copper substrate temperature profile when the lamp is in the focal plane, and a comparison between spatial uniformity ($I_{D}/I_{G}$) of graphene on Cu when the lamp is in and out of the focal plane, when the heat flux profile is flattened.

### Graphene transfer process

To characterize synthesized graphene in this work, films were transferred to 50.8 mm diameter fused silica (500 ${\upmu }$m thick) and thermal oxide (300 nm SiO$_{2}$ on 270 ${\upmu }$m thick Si) circular wafers (University Wafer, Inc) to assess graphene transmissivity and sheet resistance, respectively. TEM mesh grids (Tedpella, inc) with ultra-thin carbon support (3 nm) on copper are used for electron diffraction. A wet transfer methodology was adopted in this study. At first, PMMA powder (Sigma-Aldrich) is used in an aqueous solution at 20 mg/mL concentration^47^ as support for graphene films. The aqueous solution is then spin-coated on the graphene/copper substrates at 1000 rpm for 30 s and allowed to dry in air for 24 h. The copper substrate is etched at room temperature with iron(III) chloride (FeCl$_{3}$) solution (0.5 molar concentration) for 12–24 h^48^, after which the PMMA/graphene film floats to the surface of the solution. The film is rinsed completely by replacing the solution with distilled water using a syringe. The target substrate is then placed at 30$^\circ$ underneath the floating film. The film is lowered onto the substrate by pulling out the water with a syringe. A needle positions and pins the film edge to the substrate during the transfer process. The sample is then heated at 180 $^{\circ }$C in air for 30 min to flatten the film and rinsed thoroughly in an acetone bath to dissolve PMMA/FeCl$_{3}$ residuals.

### Characterization instruments

Raman spectra are used to assess graphene quality by calculating the intensity ratio ($I_{D}/I_{G}$) of defects in the lattice to sp2 carbon structure due to C–C in-plane vibrations^49^. A custom-built Raman microscope equipped with 40 mW excitation laser at 532 nm, 40$\times$ objective lens, and integrated with an imaging spectrometer (Horiba Ltd, iHR550) is employed for $I_{D}/I_{G}$ measurements of graphene atop copper. The spectrometer contains a 2400 g/mm blazed holographic grating and Synapse plus CCD camera (Horiba Ltd, SYN-PLUS). Graphene film transmissivity is estimated using a calibrated photodiode detector that converts 532 nm laser optical power to electrical current. A Renishaw inVia Raman microscope at the UCLA MSE department equipped with 5 mW excitation laser at 488 nm, 50$\times$ objective lens, and 1200 g/mm grating is used for Raman mapping.

Graphene sheet resistance is measured using 4-point probes (CDE ResMap 178) with 100 ${\upmu }$m tip radius, 1 mm spacing, and 100 g force at the Nanolab in the California NanoSystems Institute (CNSI). Graphene film uniformity and characteristic sizes are studied by backscattered electron (BSE) using a Zeiss Super VP40 scanning electron microscope (SEM) at the Electron Imaging Center for NanoMachines (EICN) at CNSI. Graphene hexagonal lattice structure, grain boundaries, plane spacings, and the number of layers and orientation are analyzed using electron diffraction by a FEI T12 transmission electron microscope (TEM) at 80 keV at EICN.

## Design of experiments and objective function optimization

### Experimental procedures

**Figure 2:**
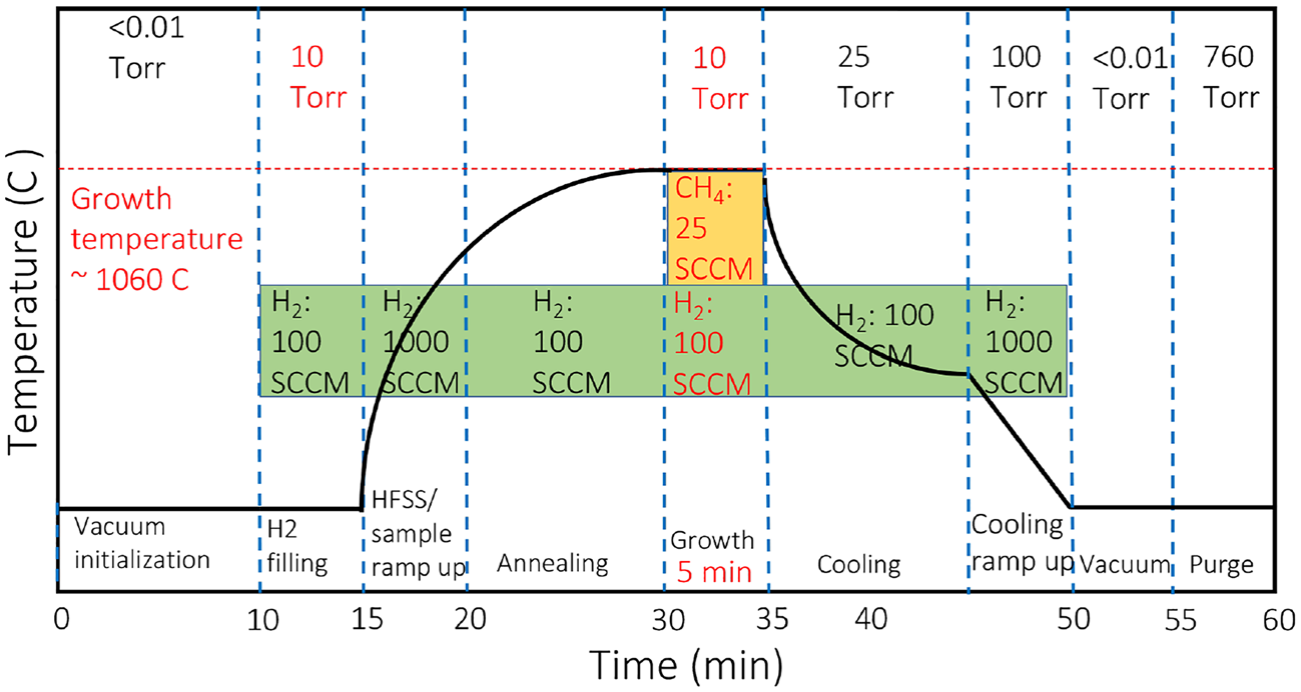
Diagram that summaries the experimental procedures, conditions and duration carried out for graphene synthesis.

A typical graphene synthesis growth process is illustrated in Fig. 2, which shows the experimental durations and conditions of each step.

At first, the copper sample (99.9% C11000 alloy foil, Revere Copper Products Inc.) is loaded into the reactor, and vacuum initialization is established to achieve a vacuum pressure of 10$^{-3}$ torr. Thereafter, reactor filling with hydrogen is initiated to reach the pressure required for graphene synthesis. Based on the desired sample temperature at various H$_{2}$ gas pressures, the HFSS total power with the corresponding current setpoint is predicted from a numerical thermal model^46^. Ramping up the solar simulator is essential to ensure that the lamp reaches its steady-state condition, which takes about 15 min^44^. Additionally, annealing the sample removes oxidation from copper and provides mechanical annealing and softening to the copper substrate. A pyrometer then acquires the temperature of the sample prior to graphene synthesis.

Graphene growth is established by introducing methane flow and setting the CH$_{4}$:H$_{2}$ gas ratio for a specific growth time. Once graphene synthesis is completed, the HFSS is switched off, and the sample is allowed to cool by increasing H$_{2}$ gas pressure gradually to 25 torr. The cooling of the sample is accelerated by increasing H$_{2}$ flow to 1000 sccm, allowing the reactor pressure to reach 100 torr. The reactor is then evacuated to remove H$_{2}$ gas residuals while ensuring the substrate temperature is below 50 $^{\circ }$C. Finally, the reactor is purged with N$_{2}$ gas to atmospheric pressure, and the sample is removed. The overall graphene synthesis process takes about 60 min, and the variable design parameters in this study are denoted in red in Fig. 2. The process parameters with their limits are shown in Table 1, and the gas composition is constrained as: $\textrm{H}_{2}(\%)+\textrm{CH}_{4}(\%) =100 \%$.

**Table 1:** Design of experiment parameters with lower and upper limits.

Parameters	Lower limit	Upper limit
Temperature ($^{\circ }$C)	1000 $^{\circ }$C	1075 $^{\circ }$C
Pressure	5 torr	50 torr
Methane (CH_4_)	10%	50%
Hydrogen (H_2_)	50%	90%
Time (min)	1 min	10 min
HFSS current (A)	100 A	115 A

### Probabilistic design of experiments (DoE)

A probabilistic surrogate model is developed to find the conditions that minimize $I_{D}$/$I_{G}$. This problem is a stochastic single-objective optimization with a Bayesian global optimization design strategy that does not assume any form for the fitted function. The process parameters taken into consideration are the copper substrate temperature, vacuum pressure, CH$_{4}$:H$_{2}$ gas ratio, and time.

A Gaussian process (GP) is implemented by creating a multivariate Gaussian distribution of the four parameters above^40^:

2 $$\begin{aligned} f(\cdot ) \sim \text{ GP }\left( m(\cdot ), k(\cdot , \cdot ) \right) \end{aligned}$$

where $m:R^d \rightarrow R$ is the mean function and $k:R^d \times R^d \rightarrow R$ is the covariance function. $\textbf{x}_{1:n}=\{\textbf{x}_1,\dots ,\textbf{x}_n\}$ represents *n* points in $R^d$, and $\textbf{f}\in R^n$ represents the outputs of $f(\cdot )$ on each element of $\textbf{x}_{1:n}$:

3 $$\begin{aligned} \textbf{f} = \left( \begin{array}{c} f(\textbf{x}_1)\\ \vdots \\ f(\textbf{x}_n) \end{array} \right) . \end{aligned}$$

The presence of $f(\cdot )$ as a Gaussian process with mean function $m(\cdot )$ and covariance function $k(\cdot , \cdot )$ implies that the vector of outputs $\textbf{f}$ at arbitrary inputs within $\textbf{X}$ follows a multivariate normal distribution:

4 $$\begin{aligned} \textbf{f} | \textbf{x}_{1:n}, m(\cdot ), k(\cdot , \cdot ) \sim \mathcal {N}\left( \textbf{m}(\textbf{x}_{1:n}), \textbf{K}(\textbf{x}_{1:n}, \textbf{x}_{1:n}) \right) \end{aligned}$$

with mean vector:

5 $$\begin{aligned} \textbf{m}(\textbf{x}_{1:n}) = \left( \begin{array}{c} m(\textbf{x}_1)\\ \vdots \\ m(\textbf{x}_n) \end{array} \right) \end{aligned}$$

and covariance matrix:

6 $$\begin{aligned} \textbf{K}(\textbf{x}_{1:n},\textbf{x}_{1:n}) = \left( \begin{array}{ccc} k(\textbf{x}_1,\textbf{x}_1) &{} \dots &{} k(\textbf{x}_1, \textbf{x}_n)\\ \vdots &{} \ddots &{} \vdots \\ k(\textbf{x}_n, \textbf{x}_1) &{} \dots &{} k(\textbf{x}_n, \textbf{x}_n) \end{array} \right) . \end{aligned}$$

Taking $\textbf{x}_{1:n}=\{\textbf{x}_1,\dots ,\textbf{x}_n\}$ as the input and *f* as the output ($I_{D}$/$I_{G}$), $f(\cdot )$ is evaluated on each of the elements of $\textbf{x}_{1:n}$. The method incorporates the use of the Matern 52 kernel function as follows:

7 $$\begin{aligned} k(r)=\sigma ^{2}\left( 1+\sqrt{5} r+\frac{5}{3} r^{2}\right) \exp (-\sqrt{5} r) \end{aligned}$$

where $r=\sqrt{\sum _{i=1}^{d} \frac{\left( x_{i}-y_{i}\right) ^{2}}{\ell _{i}^{2}}}$, $\rho$ is the lengthscale (4 parameters), $\eta$: is the variance, $\sigma _n$ is Gaussian noise variance.

Using Bayes’ rule, the posterior probability metric over the space of functions is defined as^40^:

8 $$\begin{aligned} p(f(\cdot )|\mathcal {D}) \propto p(\mathcal {D}|f(\cdot )) p(f(\cdot )) \end{aligned}$$

where $\mathcal {D}$ is the data, $p(f(\cdot ))$ is the prior, $p(f(\cdot )|\mathcal {D})$ is the posterior, and $p(\mathcal {D}|f(\cdot ))$ is the likelihood of the data. The objective function being optimized in this algorithm is defined as:

9 $$\begin{aligned} \textbf{x}^* = \arg \min _{\textbf{x}}f(\textbf{x}). \end{aligned}$$

**Figure 3:**
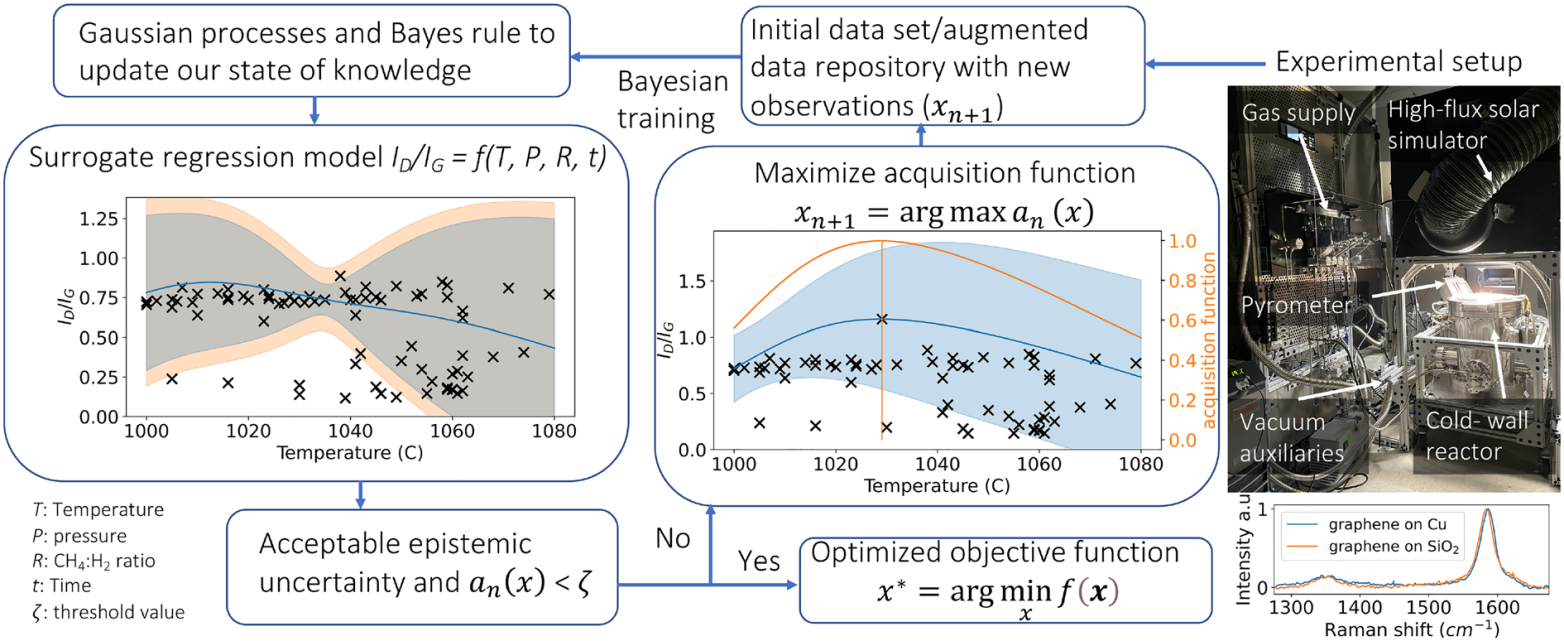
Process diagram for the design of experiment (sequential information acquisition algorithm and objective function optimization).

The algorithm starts with an initial data set consisting of input–output observations that is used to quantify the state of knowledge about $f(\textbf{x})$, and the Gaussian process is updated to obtain a predictive distribution as shown in Fig. 3.

The probabilistic surrogate model in this work utilizes epistemic uncertainty (deficiencies due to limited knowledge and uncertainties in measurements due to manufacturing imperfections and operation) to define an information acquisition function. The expected improvement (EI) over the dominant hypervolume, a common form of acquisition function, is implemented to handle measurement noise and parametric uncertainties^40,41^ as follows:

10 $$\begin{aligned} EI_n(\textbf{x}) = \frac{m_n(\textbf{x}) - m_n^*}{\sigma _n(\textbf{x})}\Phi \left( \frac{m_n(\textbf{x}) - m_n^*}{\sigma _n(\textbf{x})}\right) + \phi \left( \frac{m_n(\textbf{x}) - m_n^*}{\sigma _n(\textbf{x})}\right) . \end{aligned}$$

where $m_n(\textbf{x})$ and $\sigma _n(\textbf{x})$ are the predictive mean and variance; $m_n^*$ is the best observed minimum; $\Phi$ and $\phi$ are the cumulative distribution function and probability density function of the standard normal.

By maximizing an acquisition function ($a_{n_0}(\textbf{x})$) that depends on the current state of knowledge, the most important point is picked and evaluated^40^ $\textbf{x}_{n+1} = \arg \max a_{n}(\textbf{x})$. The function is evaluated at $\textbf{x}_{n+1}$, where function evaluation is executed by conducting a new experimental set. The original data set is then augmented by new observations, where Bayes’ rule is used to update the state of knowledge. Thus, the acquisition function helps to quantify the methodology of evaluating the objective function with new parameters by solving this process iteratively until acceptable epistemic uncertainty or a threshold value is achieved.

## Results and discussion

### Surrogate Gaussian process regression model with Bayesian optimization

The BO design strategy with GPR minimizes the objective function $I_{D}/I_{G}$ and finds the optimum parameters for graphene growth (temperature, vacuum pressure, CH$_{4}$:H$_{2}$ ratio, and growth time). Raman measurements from experimental sets update the state of knowledge of the GPR model and are acquired directly from synthesized graphene on copper for ease of processing. Figure 3 illustrates Raman spectra to compare graphene D and G peaks acquired from atop of the copper substrate to those from graphene transferred to fused silica. The results indicate that the acquired Raman spectra are not affected by interference from the copper substrate for graphene D and G peaks.

With the xenon arc lamp placed at the focal point of a truncated ellipsoidal reflector and the copper substrate at the target focal plane, the heat flux on the substrate is most concentrated. Figure 1c illustrates the copper substrate temperature profile at a HFSS current of 107 A and hydrogen gas pressure of 10 torr (prior to deposition), producing temperature of 1060 $^{\circ }$C within a circular area of 12 mm in diameter. The thermal model^46^ predicts the substrate’s temperature (measured later by a pyrometer) at various conditions and thus facilitates conducting experiments to optimize the objective function $I_{D}/I_{G}$.

**Figure 4:**
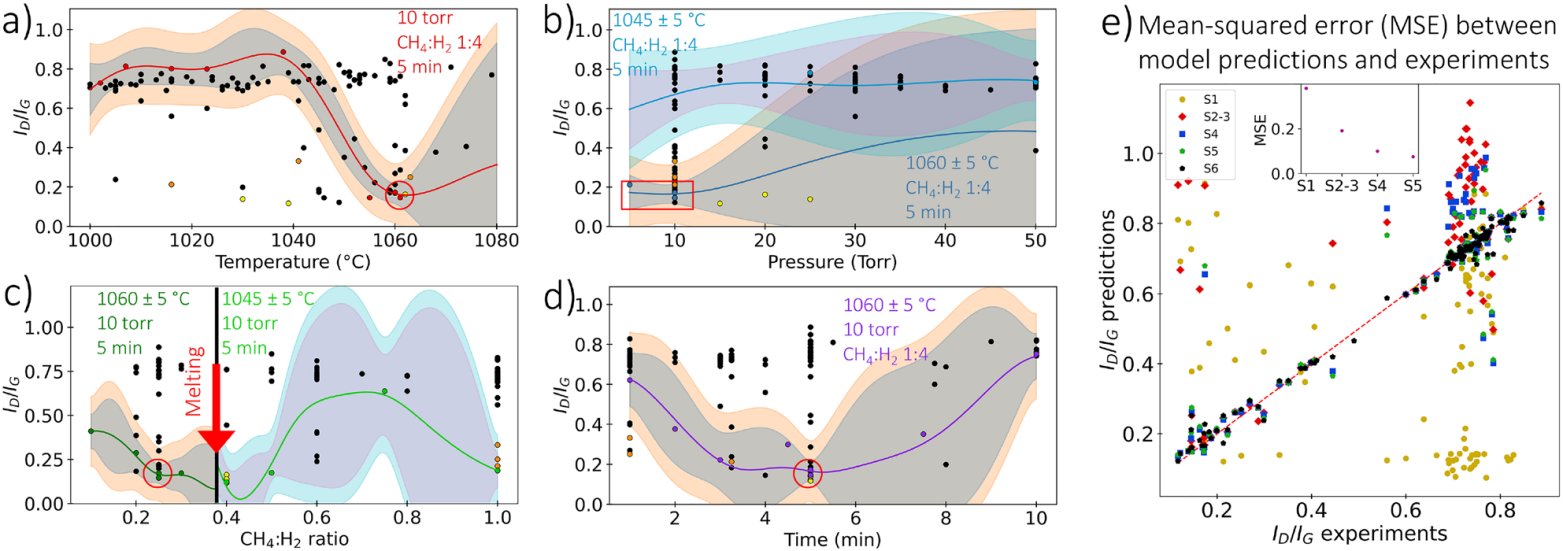
The objective function $I_{D}/I_{G}$ from the GPR-BO model as a function of (**a**) temperature, (**b**) pressure, (**c**) CH$_{4}$:H$_{2}$ ratio, and (**d**) time by varying one parameter with the others held constant, where only the experimental points denoted with the same color of the fitted surrogate model curve belong to that parametric space. The red circle denotes the same optimized condition viewed from a different spatial perspective. (**e**) Mean-squared error (MSE) between the $I_{D}/I_{G}$ predicted by the model and observed from experiments, showing the MSE and improvement of the model’s prediction of the 109 conditions after updating its state of knowledge with new sets.

The DoE starts with an initial set of experiments (Set 1) carried out systematically by varying one design parameter within the ranges shown in Table 1, with others held constant. Sets 2–3 were carried out under various conditions by inspecting the GPR-BO model and conducting the experiment in spaces that lack observations, where each initial set consists of 20 experiments. Sets 4–6, each with 15 experiments, were carried out based on the algorithm and suggested experiments that maximize the acquisition function. The mean-squared error (MSE) between the model predicted objective function ($I_{D}/I_{G}$) and experimental observations is shown in Fig. 4e. The MSE shows pronounced improvement in model predictions of the 109 different conditions after iteratively updating its state of knowledge with new observations. The optimal parameters suggested by the Bayesian Optimization (BO) in this case are considered the best conditions found within our budget, given that the objective function is highly expensive and time-consuming. The systematic approach of GPR-BO proved invaluable in exploring diverse parametric spaces and identifying conditions for the production of single-layer graphene, underscoring the method’s effectiveness alongside fundamental physics in achieving high-quality outcomes.

The final results for the objective function $I_{D}/I_{G}$ from the GPR-BO model are illustrated in Fig.  4 as a function of (a) temperature, (b) pressure, (c) CH$_{4}$:H$_{2}$ ratio, and (d) time, where each parameter is varied while others are held constant. Due to the projection of a 5-dimensional space onto a 2-dimensional plot, black, yellow, and orange experimental points do not belong to these spaces; they only share the main variable parameter on the x-axis and $I_{D}/I_{G}$ on the y-axis. The optimized conditions from the GPR-BO model are a temperature of 1060 $^{\circ }$C, gas ratio CH$_{4}$:H$_{2}$ of 1:4, and growth time of 5 min with vacuum pressure of 5 torr for SLG and 10 torr for AB-stacked bilayer graphene. The red circle denotes the same optimized condition viewed from a different spatial perspective.

### Characterization of different conditions

BSE images and Raman mapping of graphene on Cu and transferred thermal oxides, respectively, were used to assess the effects of different conditions on preferential graphene characteristic sizes within certain crystallographic orientations of Cu^50^, film quality, and uniformity. The presence of photoluminescence, peaking at 593 nm at room temperature^51^, adds complexity to the characterization process on copper and Si/SiO$_{2}$ compared to graphene on dielectric materials such as SiO$_{2}$/Si^12^, causing interference in the background signal. The DoE results indicate that higher temperature leads to high graphene quality, as illustrated in Fig. 4a. Kim et al.^25^ attribute graphene synthesis to the crystallization of a supersaturated carbon-adatom species. The nucleation density is governed by phenomena that vary with temperature, with activation energy between 1 and 3 eV. The decrease in nucleation density (larger grain size) with increasing temperature is attributed to an increase in the probability of capturing supercritical carbon nuclei from the gas feed over initiating nucleation on newly available substrate sites because of increased desorption rates^25^. In addition, high temperature enhances dissociation of carbon precursor (CH$_{4}$) as well as enlarging copper grain sizes while smoothing its surface^32^.

**Figure 5:**
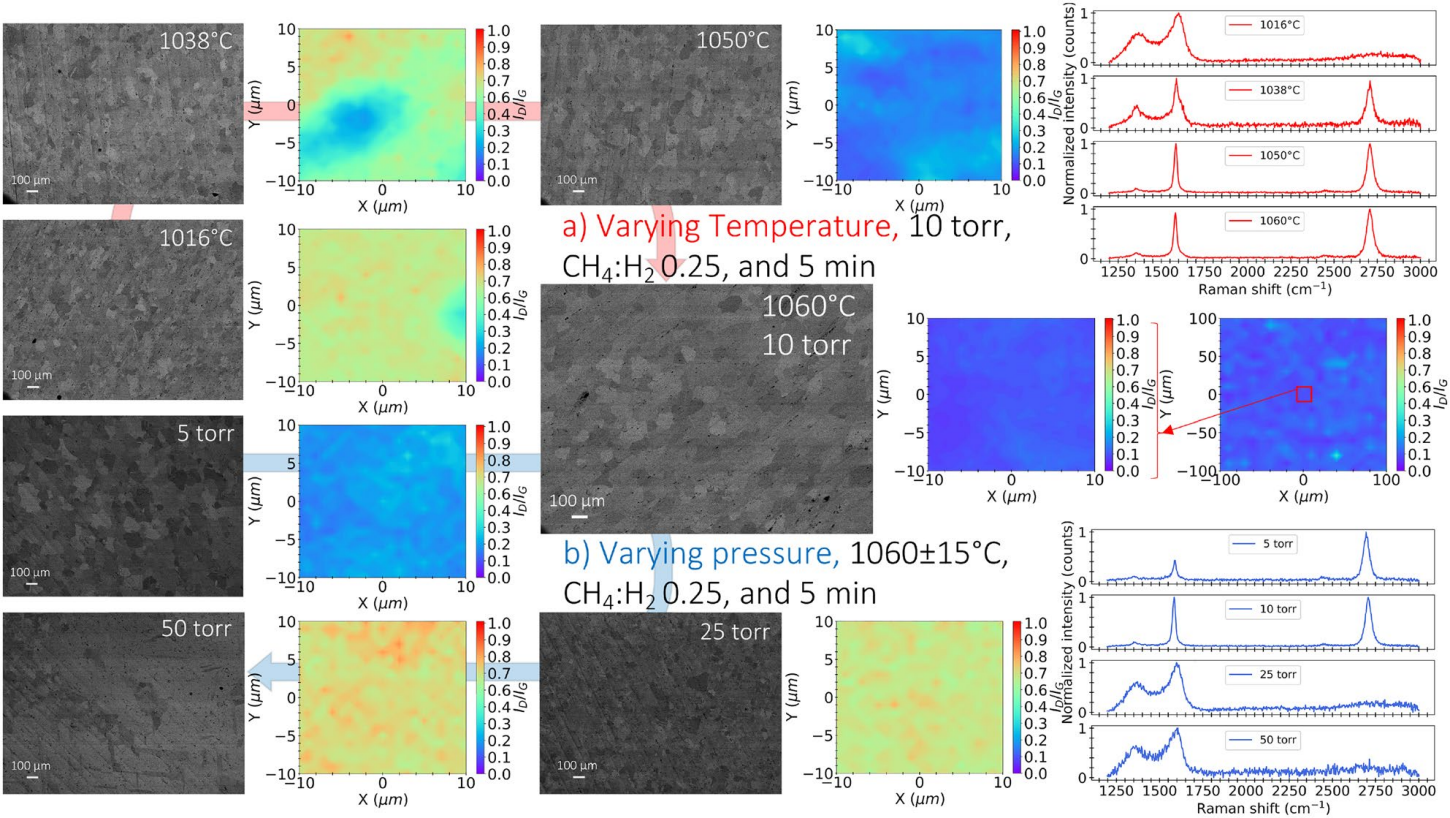
BSE images and Raman mapping that show graphene characteristic sizes and film uniformity from Raman measurements ($I_{D}/I_{G}$) synthesized at different (**a**) temperatures (1016, 1038, 1050, and 1060 $^{\circ }$C), while other parameters are held at (10 torr, CH$_{4}$:H$_{2}$ 0.25, and 5 min) and (**b**) pressure (5, 10, 25, and 50 torr), while other parameters are held at (1060 $^{\circ }$C, CH$_{4}$:H$_{2}$ 0.25, and 5 min). Optimized graphene shows Raman uniformity ($I_{D}/I_{G}$) over a characteristic length of 20 and 200 ${\upmu }$m.

Figure 5a shows BSE images and Raman mapping of graphene synthesized at 1016, 1038, 1050, and 1060 $^{\circ }$C, respectively, with other parameters are held at (10 torr, CH$_{4}$:H$_{2}$ 0.25, and 5 min). Nanocrystalline graphene’s Raman signature^49^ is dominant at the lowest temperature (1000 $^{\circ }$C), with high defects and small characteristic sizes from BSE images of graphene on Cu. In contrast, high-quality graphene growth is observed at higher temperatures (1060 $^{\circ }$C) with large characteristic size. At 1060 $^{\circ }$C, high-quality^52^ uniform graphene is achieved with $I_{D}/I_{G}$ of 0.11 and characteristic sizes of 100 ${\upmu }$m, respectively, compared to lower-quality graphene at 1016 $^{\circ }$C with 0.62 and 20 ${\upmu }$m.

Low pressure leads to high-quality graphene, as shown in Fig. 4b. In general, the cooling rate, geometry of the reactor, and pressure are important factors in graphene growth kinetics, density of defects, and thickness uniformity^26^. At low pressures, growth is limited by the surface reaction regime, which is highly sensitive to temperature uniformity. The flux of active species is reduced in the low-pressure regime, leading to fewer collisions and enhanced diffusion through the boundary layer, as explained by Bhaviripudi et al.^26^. By increasing pressure, diffusion through the boundary layer becomes the limiting factor, where the geometry of the reactor and gas flow affects the thickness of the boundary layer, leading to non-uniform graphene^26^.

Figure 5b shows BSE images and Raman mapping of graphene synthesized at 5, 10, 25, and 50 torr, respectively, with other parameters are held at (1060 $^{\circ }$C, CH$_{4}$:H$_{2}$ 0.25, and 5 min). At low pressures (5–10 torr), high-quality graphene is achieved with $I_{D}/I_{G}$ = 0.11–0.21 and characteristic sizes up to 100 ${\upmu }$m. Based on Raman results, 5 and 10 torr favor the growth of SLG and AB-stacked graphene, respectively. Conversely, high pressure leads to deterioration of graphene quality ($I_{D}/I_{G}$ = 0.68) with discontinuous films even at slightly reduced temperature from 1060 to 1045 $^{\circ }$C as shown in Fig. 5b.

High hydrogen concentrations (low CH$_{4}$:H$_{2}$ ratios) in graphene growth produce high-quality films as presented in Fig. 4c. Hydrogen has a double role that facilities methane chemisorption as an etching reagent that controls the size and morphology of graphene grains and as an activator of surface-bound carbon as demonstrated by Vlassiouk et al.^27^. Hydrogen also counteracts the negative impact of oxidizing contaminants in copper foil or stray oxygen in the gas feed during synthesis^27^. Additionally, the decrease in methane partial pressure (concentration) leads to lower nucleation density^25^.

**Figure 6:**
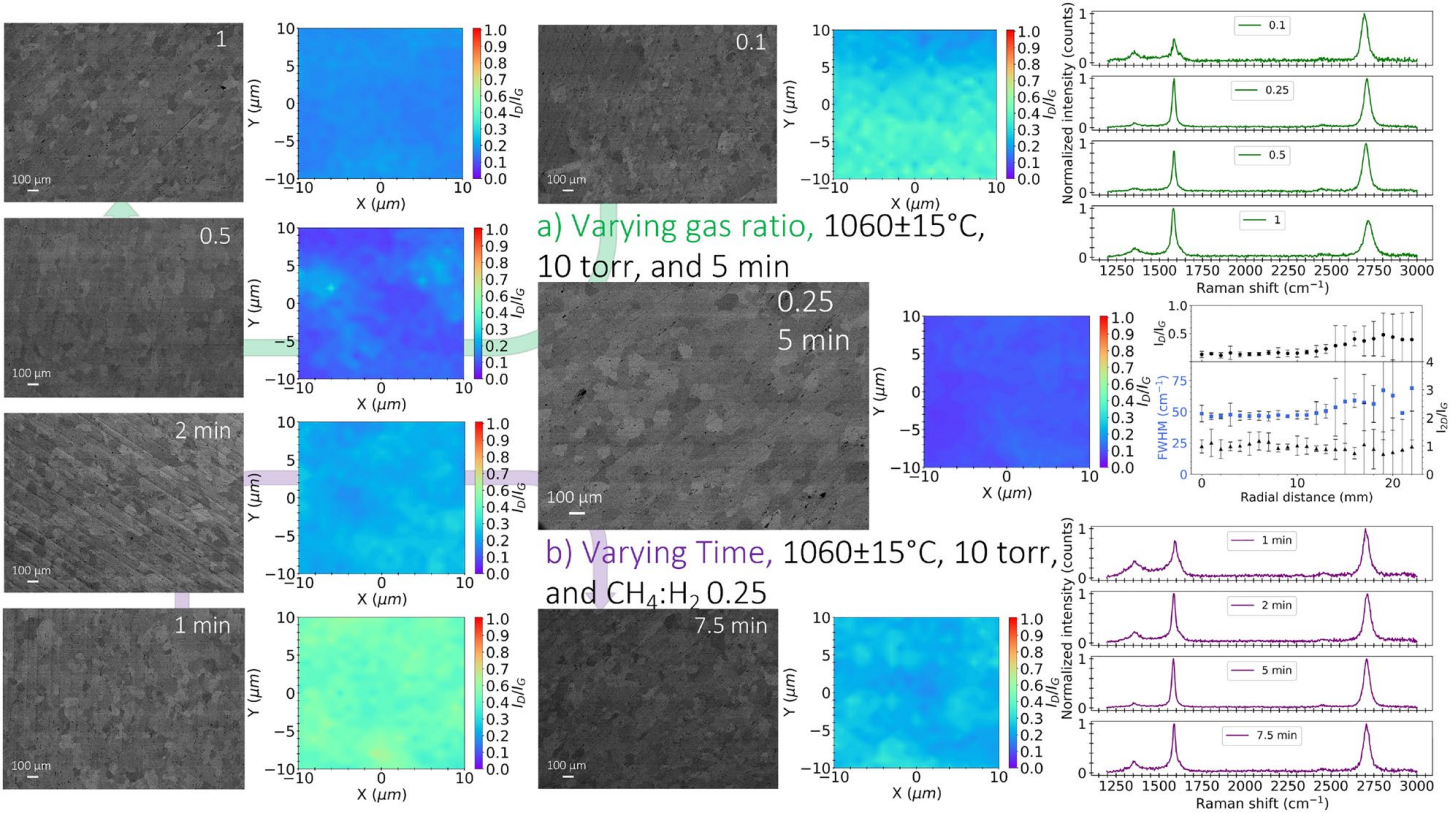
BSE images and Raman mapping that show graphene characteristic sizes and film uniformity from Raman measurements ($I_{D}/I_{G}$) synthesized at different (**a**) CH$_{4}$:H$_{2}$ ratio (0.1 0.25, 0.5, and 1), while other parameters are held at (1060 $^{\circ }$C, 10 torr, and 5 min) and (**b**) time (1, 2, 5, and 7.5 min), while other parameters are held at (1060 $^{\circ }$C, 10 torr, and CH$_{4}$:H$_{2}$ 0.25). Optimized graphene shows Raman uniformity ($I_{D}/I_{G}$, $I_{2D}/I_{G}$, and FWHM(2D)) with spatial uniformity up to 13 mm radius. Error bars show the uncertainty of radial measurements at an increment of 1 mm with four different angular positions (0$^{\circ }$, 90$^{\circ }$, 180$^{\circ }$, and 270$^{\circ }$).

Figure 6a shows BSE images and Raman mapping of graphene synthesized at 0.1, 0.25, 0.5, and 1 CH$_{4}$:H$_{2}$ ratios, respectively, with other parameters are held at (1060 $^{\circ }$C, 10 torr, and 5 min). A CH$_{4}$:H$_{2}$ ratio of 0.25 is the optimized condition with $I_{D}/I_{G}$ = 0.11, where smooth edges and large characteristic sizes up to 100 ${\upmu }$m are observed. Further reduction of the gas ratio to 0.1 produces a SLG signature and worsens graphene quality ($I_{D}/I_{G}$ = 0.41) with small characteristic sizes and discontinuous/nonuniform film coverage, as shown in Fig. 6a.

Synthesis at a higher gas ratios ($\ge$ 0.4) leads to the melting of copper if the synthesis is performed at temperatures around 1060 $^{\circ }$C (close to copper’s melting point), and for long durations (> 2 min) as shown in Fig. 4c. The increase in temperature of the sample during the growth is due to the significant reduction in hydrogen partial pressure in the chamber by introducing a higher concentration of CH$_{4}$ gas, where hydrogen’s influence as a cooling agent with higher thermal conductivity is reduced. To achieve synthesis at higher CH$_{4}$:H$_{2}$ ratios, the temperature was reduced to 1045 $^{\circ }$C prior to growth. However, the synthesis starts to follow a transient thermal behavior in which temperature changes during graphene growth. Excessive methane supply leads to non-uniform graphene films and the growth of a large number of layers despite the observed low $I_{D}/I_{G}$ ratio with CH$_{4}$:H$_{2}$ ratio of >0.5. The growth process for CH$_{4}$:H$_{2}$ ratios in the range 0.5–1 involves the adverse effect of increased CH$_{4}$:H$_{2}$ ratio (increased nucleation density) and the positive effect of high temperature close to the melting point. According to Xing et al.^29^, the carbon consumed during nucleation decreases as temperature increases, favoring the growth of single layer graphene with larger island sizes. Thus, the reason that the quality of graphene improves towards CH$_{4}$:H$_{2}$ ratio of 1 might be attributed to increases in sample temperature during the synthesis due to decreased hydrogen content, and C consumption during nucleation decreases even though the concentration of CH$_{4}$:H$_{2}$ is high, leading to high graphene quality.

Growth time is a unique feature associated with the solar-thermal CVD due to the effect of heat flux impingement on the surface and the direct rapid heating of the substrate in a short time, with the walls kept cool. Figure 4d shows that between 3.5 and 5.5 min, graphene synthesis produces a similar quality and continuous films. Figure 6b shows BSE images and Raman mapping of graphene synthesized at 1, 2, 5, and 7.5 min, respectively, with other parameters are held at (1060 $^{\circ }$C, 10 torr, and CH$_{4}$:H$_{2}$ 0.25). At 5 min, high-quality graphene is achieved with averaged $I_{D}/I_{G}$ = 0.11 and characteristic sizes up to 100 ${\upmu }$m, whereas lower and higher synthesis durations of 1 and 7.5 min show $I_{D}/I_{G}$ of 0.47 and 0.26, respectively.

The yellow and orange points in Fig. 4 represent good conditions but do not belong to these spaces. The yellow points correspond to the synthesis of graphene at higher vacuum pressure between 15 and 25 torr and gas ratio CH$_{4}$:H$_{2}$ of 0.4 with a temperature of 1045 $^{\circ }$C for 5 min. The results show macroscopically non-uniform but high-quality graphene films. As noted earlier, increasing pressure and methane concentration adversely affect graphene uniformity by introducing a non-uniform boundary layer and excessive supply of methane, respectively, which lead to less uniform graphene films. The orange points correspond to the synthesis of graphene at a vacuum pressure of 10 torr and gas ratio of CH$_{4}$:H$_{2}$=1. Synthesis occurred either at a higher temperature of 1060 $^{\circ }$C for a short time of 1 min or a lower temperature of 1000 $^{\circ }$C with a longer duration of 3 min, leading to high graphene quality with $I_{D}/I_{G}<$ 0.25. The short-time result is attributed to the fact that the partial pressure of methane remains low within one minute of growth as it mixes with higher hydrogen concentration (actual CH$_{4}$:H$_{2}<$ 1), which explains the high graphene quality despite the gas ratio set point and without melting the sample. The 3-min growth behavior is attributed to transient thermal effects arising from the abrupt changes in temperature during synthesis that counteracts the adverse effect of high CH$_{4}$:H$_{2}$ as discussed earlier. Table 2 shows the averaged product metrics, $I_{D}/I_{G}$, $I_{2D}/I_{G}$, and FWHM(2D) from Raman mapping, of the different experimental conditions.

### Characterization of graphene with single and AB-stacked bilayer Raman signatures

**Figure 7:**
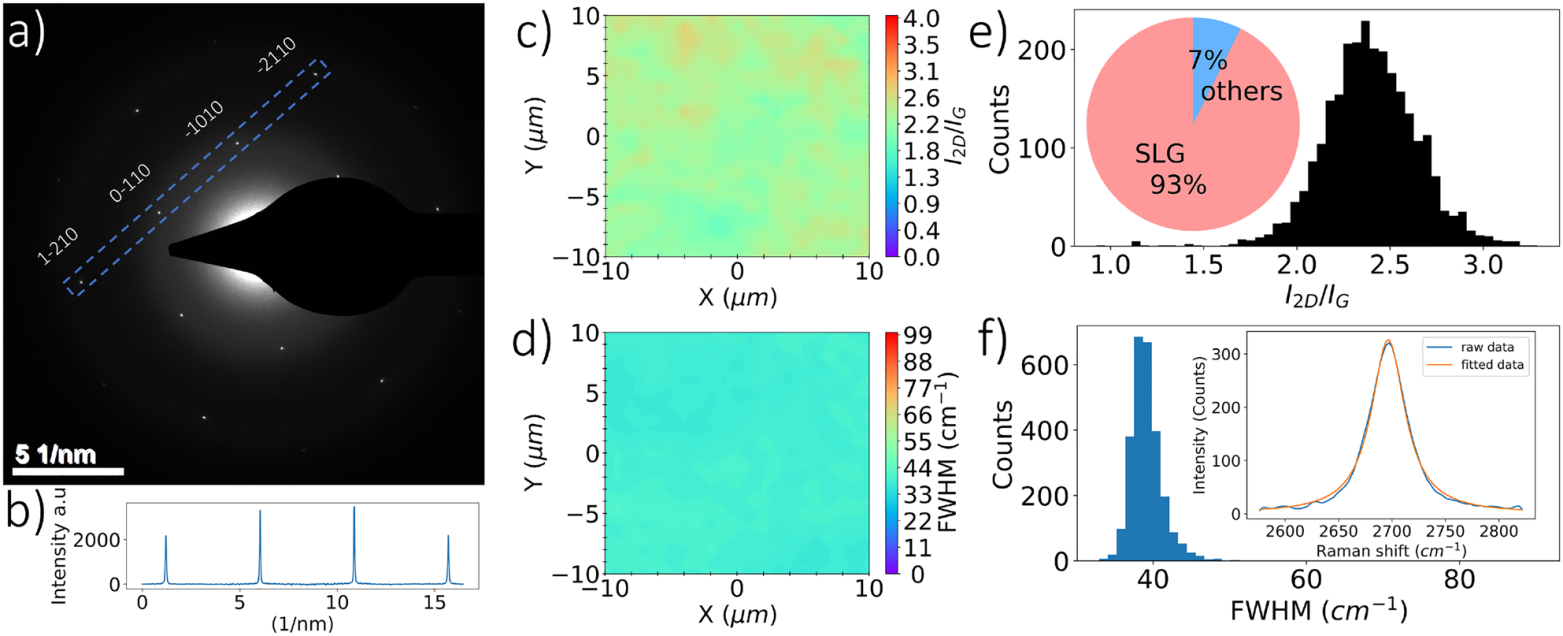
Single-layer graphene characterization: (**a**) electron diffraction from a single graphene grain of 5 ${\upmu }$m (aperture size). (**b**) Intensity profile within the blue dashed box in part (**a**) showing outer peaks of equivalent [2100] planes and inner peaks from equivalent [1100] planes. Raman mapping illustrates uniformity of (**c**) $I_{2D}/I_{G}$ and (**d**) FWHM(2D) over characteristic lengths of 20 ${\upmu }$m acquired from graphene on 300 nm SiO$_{2}$/Si. (**e**) Statistical distribution of $I_{2D}/I_{G}$, where the inset estimates the stacking ratio of SLG when compared to other Raman spectra of few-layer graphene. (**f**) Statistical distribution of FWHM(2D), where the inset shows the 2D Lorentzian fit of SLG.

**Figure 8:**
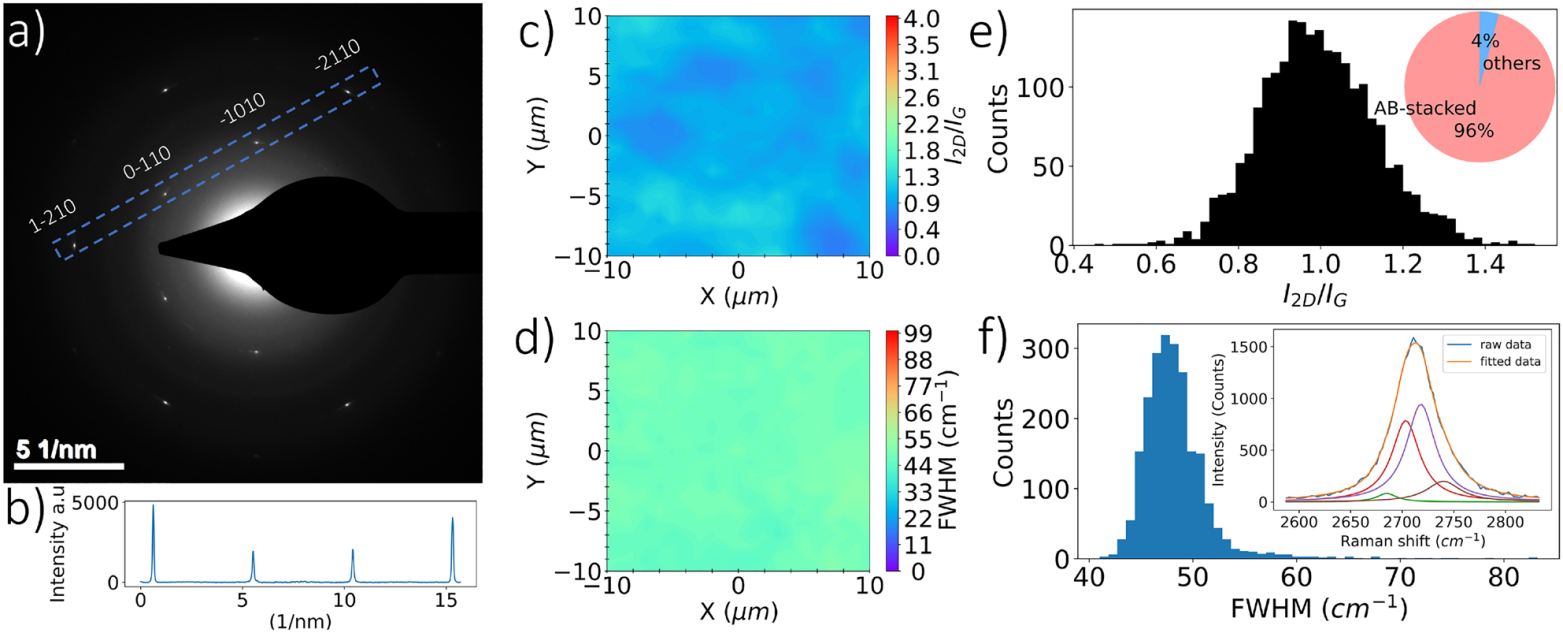
AB-stacked bilayer graphene characterization: (**a**) electron diffraction from a single graphene grain of 20 ${\upmu }$m (aperture size). (**b**) Intensity profile within the blue dashed box in part (**a**) showing outer peaks of equivalent [2100] planes and inner peaks from equivalent [1100] planes. Raman mapping illustrates uniformity of (**c**) $I_{2D}/I_{G}$ and (**d**) FWHM(2D) over a characteristic length of 20 ${\upmu }$m acquired from graphene on 300 nm SiO$_{2}$/Si. (**e**) Statistical distribution of $I_{2D}/I_{G}$, where the inset estimates the stacking ratio of AB-stacked bilayer graphene when compared to other Raman spectra of few-layer graphene. (**f**) Statistical distribution of FWHM(2D), where the inset shows the 2D Lorentzian fit of AB-stacked bilayer graphene.

After optimizing graphene synthesis with conditions that produce high-quality SLG and AB-stacked bilayer graphene, further characterization was carried out to assess lattice structure, number of layers and orientation, film transmissivity, and sheet resistance. Electron diffraction analysis of graphene on TEM grids shows different patterns that vary from a single grain to polycrystalline graphene^53^. Synthesis of graphene on polycrystalline Cu that contains different grain sizes and surface conditions affects graphene nucleation density, grain sizes, and boundary shapes^25^. In the foregoing study, graphene synthesis on commercial polycrystalline Cu, a material that has not undergone any surface polishing, was explored. This material contained varying grain sizes and surface conditions, which affects graphene nucleation density, grain sizes, and boundary shapes, as highlighted in previous research^25^. Therefore, electron diffraction for a single grain of SLG and AB-stacked bilayer graphene with 5 ${\upmu }$m and 20 ${\upmu }$m sizes (aperture sizes), respectively, were used to characterize synthesized graphene films as shown in Figs. 7a and 8a. The difference between graphene grain sizes evaluated by electron diffraction and characteristic sizes by BSE analysis of graphene on Cu can be attributed to the formation of multiple smaller graphene islands (grains) due to increased nucleation density^25^ within certain crystallographic orientations of copper^50^, as well as induced wrinkles, overlaps, and adlayers during the transfer process^47,54,55^.

The hexagonal lattice patterns of graphene from electron diffraction analysis illustrate plane spacings d$_{11}$, and d$_{10}$ of 0.12, and 0.21 nm, respectively, and a d$_{10}$/d$_{11}$ ratio of about $\sqrt{3}$. Thus, the lattice constant ($\tilde{a}$) for graphene is approximately 0.14 nm. Diffraction intensity ratios labeled by Bravis–Miller indices from outer peaks of equivalent planes [1–210] to inner peaks of equivalent planes [1–100] ($I_{1-210}$/$I_{0-110}$ and $I_{-2110}$/$I_{-1010}$) are approximately 0.5 for monolayer graphene and 2 for AB-stacked bilayer graphene^12,13,56^. Figures 7b and 8b show diffraction intensities near 0.5 for SLG and 2 for AB-stacked bilayer graphene, respectively. Statistical SAED patterns, obtained by examining a minimum of five different locations, are provided in Table S1 in Supplemental Information. Due to the elaborate and costly nature of acquiring extensive electron diffraction data for statistical analysis, Raman mapping was utilized as an alternative characterization method to assess the repeatability of the graphene signature.

Raman mapping was conducted on graphene transferred to SiO$_{2}$/Si to characterize graphene coverage and number of layers statistically. The Raman spectrum of SLG exhibits distinctive features, and the 2D peak is symmetric with FWMH and $I_{2D}/I_{G}$ values of 34–45 cm$^{-1}$ and 1.8–2.8, respectively^13,25,26,32,33,35,57^. Figures 7c and 7d show Raman maps of $I_{2D}/I_{G}$ and FWHM(2D), respectively, carried out on a square area with 20 ${\upmu }$m characteristic length. Additionally, the distribution of FWHM(2D) and $I_{2D}/I_{G}$ of SLG at six different locations are included in Figs. 7e,f. Here, we observe 2D peak features consistent with SLG, with averaged $I_{2D}/I_{G}$ and FWHM(2D) of 2.39 and 39.2 cm$^{-1}$, respectively, and fitted with one Lorentzian as shown in the inset of Fig. 7f. Thus, the results reveal a uniform graphene film with about 93% SLG coverage based on these Raman features.

The Raman spectrum of AB-stacked bilayer graphene exhibits distinctive features as well, where the 2D peak is asymmetric with FWMH and $I_{2D}/I_{G}$ values of 40–62 cm$^{-1}$, and 0.75–1.46, respectively^12,13,56^. Additionally, AB-stacked graphene’s electronic structure can be uniquely captured with Raman spectroscopy, where a double resonant Raman process is observed, and the 2D peak is split into four components^58^. Figures 8c and 8d show Raman mappings of $I_{2D}/I_{G}$ and FWHM(2D), respectively, carried out on a squared area with 20 ${\upmu }$m characteristic length. Additionally, the distributions of FWHM(2D) and $I_{2D}/I_{G}$ of AB-stacked bilayer graphene at five different locations are included in Fig. 8e,f. The 2D peak features resemble the Raman signature of AB-stacked graphene with average $I_{2D}/I_{G}$ and FWHM(2D) of 0.996 and 48.3, respectively, and are fitted with four Lorentzian functions as shown in the inset of Fig. 8f. The results reveal a uniform graphene film with about 96% AB-stacked coverage based on these Raman features.

**Table 2:** Summary of the studied experimental conditions with averaged $I_{D}/I_{G}$, $I_{2D}/I_{G}$, and FWHM(2D) from Raman mapping with corresponding sheet resistance measurements.

Temperature ($^{\circ }$C)	Pressure (torr)	CH$_{4}$:H$_{2}$ ratio	Time (min)	$I_{D}/I_{G}$	$I_{2D}/I_{G}$	FWHM(2D) (cm$^{-1}$)	R$_{s}$ (k$\Omega$/sq)	Raman signature
1016	10	0.25	5	0.62 ± 0.10	–	–	–	–
1038	10	0.25	5	0.53 ± 0.24	–	–	363.62 ± 119.4	–
1050	10	0.25	5	0.19 ± 0.08	1 ± 0.22	47.33 ± 6.06	7.75 ± 3.9	AB-bilayer
1060	10	0.25	5	0.14 ± ± 0.04	0.98 ± 0.28	47.38 ± 3.68	3.43 ± 1.52	AB-bilayer
1060	5	0.25	5	0.21 ± 0.05	2.3 ± 0.34	38.4 ± 2.2	15.49 ± 4.64	SLG
1060	25	0.25	5	0.64 ± 0.06	–	–	51.7 ± 2.85	–
1060	50	0.25	5	0.68 ± 0.08	–	–	1941.2 ± 1364.3	–
1060	10	0.1	5	0.34 ± 0.14	2.34 ± 0.5	38.09 ± 3.52	9.09 ± 4.2	SLG
1060	10	0.5	5	0.14 ± 0.08	1.5 ± 0.42	48.71 ± 5	1.42 ± 0.18	–
1060	10	1	5	0.19 ± 0.02	0.85 ± 0.1	59.81 ± 4.7	7.98 ± 3.01	–
1060	10	0.25	1	0.47 ± 0.08	1.14 ± 0.26	52.6 ± 3.58	119.22 ± 143.89	AB-bilayer
1060	10	0.25	2	0.25 ± 0.06	1.07 ± 0.32	45.22 ± 4.02	10.04 ± 1.77	AB-bilayer
1060	10	0.25	7.5	0.26 ± 0.06	1.11 ± 0.24	48.35 ± 4.18	11.23 ± 4.73	AB-bilayer

Graphene’s optical absorptivity has been reported as $2.3 \pm 0.1\%$ per layer^4^, whereas bilayer graphene’s transmittance (AB-stacked) has been measured in previous work as 95.3% at a wavelength of 550 nm^56^. Synthesized graphene at optimized conditions were transferred to a fused silica wafer with $\tau _{quartz} = 0.93$. The measured transmissivity ($\tau$) of SLG shows values between 0.959 and 0.977, whereas bilayer graphene’s transmissivity falls in the range of 0.929–0.953. Some spots have lower values due to contamination during the wet transfer process and FeCl$_{3}$/PMMA residuals.

The nature of polycrystalline graphene with its grain boundary impairs its electrical and mechanical properties^25^. Numerous studies have explored the electronic transport characteristics of graphene. Monolayer graphene (SLG) has been observed to exhibit sheet resistances ranging from 0.65 to 0.76 k$\Omega$/sq^52^ to values as high as $10^5$ $\Omega$/sq^23^; the latter is typically associated with diminished graphene quality. Specifically, investigations on AB-stacked graphene have been conducted in previous studies^13,56^. Under zero gate voltage, these studies reported resistances within the range of 2.2–3.4 k$\Omega$ in a square channel^13,56^. Additionally, other research efforts have indicated sheet resistances as high as 9 k$\Omega$/sq for cases of lower AB-stacked coverage^12^. These studies indicate that sheet resistance is largely affected by increased $I_{D}/I_{G}$ (graphene boundary defects) and measurement length, leading to increased sheet resistance^23,32^. Conversely increased number of layers reduces sheet resistance^23,47,59^. Therefore, the sheet resistance provides another indication of graphene quality and number of layers.

In this study, graphene transferred to SiO$_{2}$/Si was assessed for sheet resistance using a four-point probe with a 1 mm spacing. Here, we report sheet resistance of optimized SLG and AB-Stacked bilayer graphene transferred to a thermal oxide wafer with values of 15.49 ± 4.64 and 3.43 ± 1.52 k$\Omega$/sq, respectively. Graphene synthesized at a higher CH$_{4}$:H$_{2}$ ratio of 0.5 produces lower sheet resistance but with a complex synthesis due to the transient process and non-uniformity in Raman signature and number of layers. Table 2 shows the averaged $I_{D}/I_{G}$, $I_{2D}/I_{G}$, and FWHM(2D) from Raman measurements at different experimental conditions with corresponding sheet resistance. Several factors can influence sheet resistance measurements when compared to values reported in prior literature. Previous studies have typically minimized probe contact resistance with graphene through metal contact deposition^12,13,56^. In this study, the supply current traverses multiple grain boundaries, affecting the observed resistance. Additionally, minor factors such as contamination from the etching process (FeCl$_{3}$), residues from copper/polymer, and the influence of thermal oxide can also contribute to variations in resistance measurements.

**Figure 9:**
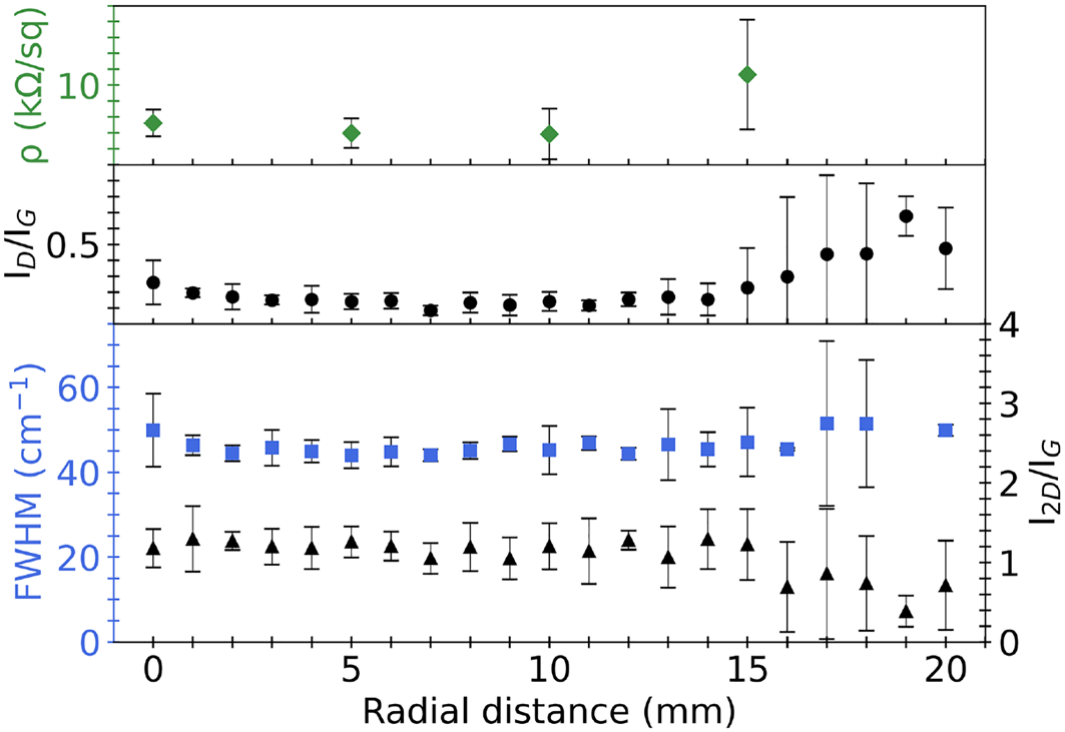
Sheet resistance of synthesized graphene transferred to thermal oxides with Raman measurements ($I_{D}/I_{G}$, $I_{2D}/I_{G}$, and FWHM(2D)) as a function of radial position, where error bars indicate the uncertainty of radial measurements at (**a**) four different angular positions (0$^{\circ }$, 90$^{\circ }$, 180$^{\circ }$, 270$^{\circ }$) with an increment of 1 mm and (**b**) co-located Raman measurements with sheet resistance at eight different angular positions (0$^{\circ }$, 45$^{\circ }$, 90$^{\circ }$, 135$^{\circ }$, 180$^{\circ }$, 225$^{\circ }$, 270$^{\circ }$, and 315$^{\circ }$) with an increment of 5 mm.

Scaled-up graphene synthesis at optimized conditions (AB-stacked bilayer) by displacing the lamp was also achieved. Figures 6 and 9 illustrate graphene spatial uniformity up to 13 mm in radius per Raman measurements of $I_{D}/I_{G}$, $I_{2D}/I_{G}$, and FWHM(2D). Additionally, co-located sheet resistance with Raman measurements indicate graphene uniformity as shown in Fig. 9. The sheet resistance measurements can have slight inconsistency due to contamination from the wet transfer and FeCl$_{3}$/PMMA residuals as well.The obtained values of AB-stacked graphene synthesized at optimized conditions, ranging from 2 to 5 k$\Omega$/sq as depicted in Fig. 9, affirm the uniformity and high quality of the graphene produced. The close alignment of larger-scale resistance measurements with reported microscale values further supports the uniformity and high quality of the graphene material. The resultant graphene film at optimized conditions was transferred onto Si/SiO$_{2}$ and fused silica wafers, as depicted in Fig. S1a,b. An optical image of graphene on Si in Fig. S1a reveals consistent contrast across the surface, suggesting a predominantly uniform graphene layer. The primary contributors to the observed contrast are residuals from copper and the etching solution. Figure S1c displays a photograph of graphene on fused silica, emphasizing a highly transmissive and clean film.

## Conclusions

This paper presents a comprehensive parametric study of graphene growth using a solar-thermal cold-wall CVD reactor to demonstrate the practicality of utilizing a renewable energy resource in synthesizing high-quality graphene films. The design of experiments adopted in this work uses a probabilistic Gaussian regression with Bayesian optimization model and an information acquisition function to quantify the parameter space and as a predictive guide in finding conditions that minimize an objective function ($I_{D}/I_{G}$). Such synthesis was conducted in a one-step process with relatively short growth times of 1–10 min. Graphene characterization shows signatures of high-quality SLG and AB-stacked bilayer graphene films through analysis conducted by electron diffraction and Raman mapping with lowest $I_{D}/I_{G}$ ratios of 0.21 and 0.14, respectively. Backscattered electron and electron diffraction analysis of SLG and AB-stacked bilayer graphene reveals grain sizes up to 5 and 20 ${\upmu }$m, respectively. The measured transmissivities of SLG and AB-stacked bilayer graphene fall in the ranges of 0.959–0.977 and 0.929–0.953, respectively, where the sheet resistance is found to be sensitive to boundary defects with values of 15.49 ± 4.64 and 3.43 ± 1.52 k$\Omega$/sq, respectively. The synthesized graphene of this work shows high-quality products and promising capabilities suitable for photonic and electronic applications. Synthesis of such graphene products can be amplified using continuous roll-to-roll processing for large-scale, sustainable production.

### Supplementary Information

Supplementary Information.

## Data Availability

An example Python code that includes representative datasets and algorithms from the present work is available in an open GitHub notebook^60^ at: https://doi.org/10.5281/zenodo.10206976. Full datasets used and/or analyzed during the current study are available from the corresponding author upon request.
