# Supplementary material for: Sequential Bayesian-optimized graphene synthesis by direct solar-thermal chemical vapor deposition
Source: Sci Rep. 2024 Feb 13;14:3660. doi: 10.1038/s41598-024-54005-z (PMC11306603; doi:10.1038/s41598-024-54005-z)
Supplement: Supplementary file 1 — Supplementary Information. [file 41598_2024_54005_MOESM1_ESM.pdf]

Supplementary Information: Sequential Bayesian-optimized graphene synthesis by direct solar-thermal chemical vapor deposition

Abdalla Alghfeli and Timothy S. Fisher*

Mechanical and Aerospace Engineering Department, University of California Los Angeles, Los Angeles, California 90095 USA

E-mail: tsfisher@ucla.edu

Number of pages: 3

Number of figures: 1

Number of tables: 1

Statistical analysis of graphene layers using SAED patterns was conducted by assessing a minimum of five distinct locations. In these locations, the graphene electron diffraction ratio was found to be approximately 0.5 for single-layer graphene and 2 for AB-stacked graphene. Due to the high cost associated with acquiring extensive electron diffraction data for statistical purposes, Raman mapping was employed as an alternative characterization method. SAED statistical analysis are provided in Table S1.

Table S1: Statistical results for Selected Area Electron Diffraction (SAED) patterns, supporting the determination of graphene layer count.

Graphene signature	#	I_{1-210}/I_{0-110}	I_{-2110}/I_{-1010}	d_{11} (nm)	d_{10} (nm)	Apertuare size (μm)
Single layer	1	0.694	0.432	0.209	0.120	5
Single layer	2	0.439	0.557	0.208	0.120	5
Single layer	3	0.649	0.591	0.205	0.120	5
Single layer	4	0.657	0.631	0.206	0.120	5
Single layer	5	0.567	0.645	0.207	0.120	5
AB-stacked bi-layer	1	2.191	2.581	0.208	0.120	20
AB-stacked bi-layer	2	2.106	1.943	0.207	0.120	20
AB-stacked bi-layer	3	1.990	1.872	0.205	0.119	20
AB-stacked bi-layer	4	2.504	1.830	0.206	0.119	20
AB-stacked bi-layer	5	2.075	1.553	0.203	0.118	20

The graphene film resulting from optimized conditions was transferred onto Si/SiO_2 and fused silica wafers, as illustrated in Fig. S1a and S1b. An optical image of graphene on Si in Fig. S1a depicts uniform contrast across the surface, indicating a predominantly uniform graphene layer. The primary contributors to the observed contrast are residuals from copper and the etching solution. Fig. S1c presents a photograph of graphene on fused silica, highlighting a highly transparent and clean film.

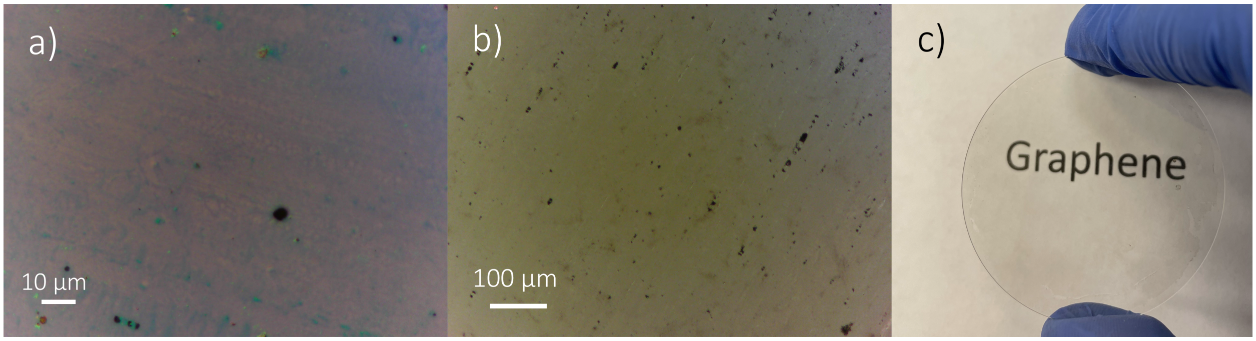

Figure S1: An optical micrograph showing graphene growth transferred on Si/300 nm SiO_2 (a) and fused silica (b). Additionally, a photograph depicting graphene film transmissivity on a fused silica wafer (c)
